# Neuromuscular mechanisms of motor adaptation to repeated gait-slip perturbations in older adults

**DOI:** 10.1038/s41598-022-23051-w

**Published:** 2022-11-18

**Authors:** Shuaijie Wang, Yi-Chung Pai, Tanvi Bhatt

**Affiliations:** Department of Physical Therapy, University of Illinois at Chicago, 1919 W Taylor St, (M/C 898), Chicago, IL 60612 USA

**Keywords:** Motor control, Neuronal physiology

## Abstract

Individuals can rapidly develop adaptive skills for fall prevention after their exposure to the repeated-slip paradigm. However, the changes in neuromuscular control contributing to such motor adaptation remain unclear. This study investigated changes in neuromuscular control across different stages of slip-adaptation by examining muscle synergies during slip training. Electromyography signals during 24 repeated slip trials in gait were collected for 30 healthy older adults. Muscle synergies in no-adaptation (novel slip), early-adaptation (slip 6 to 8), and late-adaptation trials (slip 22 to 24) were extracted. The similarity between the recruited muscle synergies in these different phases was subsequently analyzed. Results showed that participants made significant improvements in their balance outcomes from novel slips to adapted slips. Correspondingly, there was a significant increase in the muscle synergy numbers from no-adaptation slips to the adapted slips. The participants retained the majority of muscle synergies (5 out of 7) used in novel slips post adaptation. A few new patterns (n = 8) of muscle synergies presented in the early-adaptation stage to compensate for motor errors due to external perturbation. In the late-adaptation stage, only 2 out of these 8 new synergies were retained. Our findings indicated that the central nervous system could generate new muscle synergies through fractionating or modifying the pre-existing synergies in the early-adaptation phase, and these synergies produce motor strategies that could effectively assist in recovery from the slip perturbation. During the late-adaptation phase, the redundant synergies generated in the early-adaptation phase get eliminated as the adaptation process progresses with repeated exposure to the slips, which further consolidates the slip adaptation. Our findings improved the understanding of the key muscle synergies involved in preventing backward balance loss and how neuromuscular responses adapt through repeated slip training, which might be helpful to design synergy-based interventions for fall prevention.

## Introduction

The annual fall rates for older adults range from 0.3 to 1.6 times per person, with an annual cost of approximately $31 billion^1,2^. One major cause of injurious falls is slipping, which is responsible for over 40% of outdoor falls among older adults, and nearly one fifth of fractures^3^. Hence, abundant research is targeted at developing interventions for lowering the likelihood of slip-induced falls in older adults^4–8^. Among the interventions, repeated slip-perturbation training has shown to be efficacious in reducing laboratory-induced falls over longer-term^4,9,10^.

These studies have revealed that the rapid adaptation to slips occurs via improvements in both proactive and reactive control. After experiencing repeated slip perturbations, participants proactively modify their gait pattern (e.g., step length, flat foot landing, and knee flexion at heel strike), resulting in a reduction in the slip intensity^11,12^. The reactive adaptations occur in the form of a better recovery stepping location, increased magnitude of certain joint moments, and earlier onset of muscle activation^4,13^. It is known that the changes in human movement kinematics and kinetics result from motor programs generated by the CNS to signal the neuromuscular system. While changes in biomechanical variables during slip adaptation have been well analyzed and documented, the underlying neuromuscular control mechanisms that could contribute towards the movement adaptations are still unclear.

According to the muscle synergy hypothesis, the CNS simplifies motor control through the flexible combination of several muscle synergies, which are defined as a set of muscles recruited by a single neural command signal^14^. It has been proposed that the improvement in balance performance during postural perturbations is associated with spinal and supraspinal control, which are responsible for controlling the structure of muscle synergies^15,16^. Therefore, muscle synergies could be considered as the lowest level of the motor control hierarchy. Additionally, balance training could modify spinal reflex circuits and lead to persistently reduced Hoffmann-reflexes^17,18^, ultimately affecting the recruitment of muscle synergies^19^. Thus, the investigation of muscle synergies could provide new insight into the motor control mechanisms underlying perturbation-induced motor adaptation. Additionally, understanding how muscle synergies contribute to a successful recovery in the adapted trials would be helpful for development of fall prevention protocols.

Previous studies have reported that a common set of muscle synergies are used between unperturbed walking and gait perturbations, as well as across different perturbation intensities^18,20^. The use of similar muscle synergies between balance tasks indicates that common neural mechanisms might be used to maintain center of mass stability during different conditions. Therefore, it is possible that even if biomechanical adaptations occur during repeated slip training, a common set of muscle synergies could still be found between novel slips and adapted slips. However, a study comparing perturbed and unperturbed walking also reported that some muscle synergies were recruited only during the novel perturbation trial but not during unperturbed walking, and those synergies were considered to play a key role in enhancing the recovery response against the unexpected perturbation^20^. Similarly, Sawers et al. found that muscle synergies with different structures were recruited between slip-induced falls and recoveries in older adults^21^. As repeated slip training can greatly lower fall rate, it is possible that the synergies related to recoveries might be maintained and those related to falls might be discarded following repeated perturbation exposure. These changes in muscle synergies could contribute towards motor adaptation and improved reactive balance responses.

Previous evidence has shown that adaptation to repeated overground slip-perturbation exposure is induced rapidly with as few as 5–8 trials needed to reduce falls from about 50–60% to 0% in both young and older adults^22–24^. However, such early adaptation is also prone to wash out effects and/or interference, and participants have deteriorated performance when re-exposed to perturbation trials after a block of unperturbed trials or an opposing perturbation^25,26^. To induce a more long lasting adaptation, studies have used principles of motor learning to provide both block and random training and also to increase the number of trials to ensure “overtraining”^27,28^. Extensive research using single session protocols has shown that after the first block there is a plateau in adaptation in kinematic variables such as center of mass (COM) state stability, which is retained even under random training conditions (mixed slip and nonslip trials)^4,11,27^. Thus, overtraining might just help in longer-term retention (long-term gain) even if it does not result in any significant gains after the initial acquisition of adaptation (acute gain). However, given the wide range of muscle synergy patterns feasible for generating similar motor behaviors, it is possible that overtraining results in changes to muscle synergies which are not evident in biomechanical variables. In early adaptation stages, redundant muscle synergies might be used to prevent backward balance loss, which could be optimized through overtraining to minimize muscle effect (muscle activities)^29^ or motor effect cost (neuron activities)^30^. Therefore, optimization of muscle synergies through overtraining may decrease the amount of energy expenditure required to regain balance, even if biomechanical responses do not change. Thus, the changes in muscle synergies due to overtraining are not known and deserved to be analyzed.

The purpose of this study was to investigate changes in neuromuscular control during 24 repeated over-ground slips. Three stages were selected from these trials: the no-adaptation stage (novel slip (S1)), early-adaptation stage (S6–S8), and late-adaptation stage (S22–S24). We hypothesized that there would be changes in muscle synergies from novel slip to adapted slips, indicated by modifications in structure and increment in number of muscle synergies. We also hypothesized that there would only be minor changes in muscle synergy structure from early-adaptation to late-adaptation stages, as overtraining is usually included in motor leaning or training protocols for consolidation or refinement of acquired performance gains.

## Methods

### Subjects

Thirty healthy older adults (age: 72.6 ± 5.9 years, height: 1.64 ± 0.21 m, mass: 74.7 ± 11 kg, male: 11) participated in the study. They were selected from our data-base on overground slip-perturbation training containing the data of 38 participants (age: 72.1 ± 5.6 years, height: 1.66 ± 0.11 m, mass: 75.3 ± 12.2 kg, male: 14)^31^. Participants were screened via a questionnaire before the experiment to exclude individuals with any neurological, musculoskeletal, cardiopulmonary, or other systemic disorders that would make participation unsafe. These participants were selected based on the following criteria: firstly, participants with missing electromyography data (missing channels or artifacts due to motion or other sources were not included in this study) were excluded. Second, left-leg dominant participants (determined by self-report of the preferred leg for kicking a ball) were also excluded, as a previous study has reported differences in the number of muscle synergies between dominant side and non-dominant side^32^. The study was carried out in accordance with the Declaration of Helsinki of 1975, and all participants provided written informed consent which was approved by the Institutional Review Board of the University of Illinois at Chicago.

### Experimental protocol

All subjects received 24 slip trials during walking provided in a “mix-and-blocked” manner. This protocol consisted of 10 trials of unperturbed walking, a block of 8 repeated slips (S1–S8), a block of 3 nonslip trials, another block of 8 repeated slips (S9–S16), and a final block of 15 mixed trials. The mixed block contains 7 nonslip trials and 8 slip trials (S17–S24)^31^. The slip was introduced by a pair of low-friction moveable platforms (L: 0.65 × W: 0.30 m) imbedded in a 7-m walkway and hidden by stationary decoy platforms surrounding them. Thus, the sliders were less apparent to the participants. Without participants’ awareness, the right slider would be released to freely slide in the anterior–posterior direction up to 90 cm to bring the COM posterior to the base of support, just as a slip does in real-life. The instruction was consistent for all trials as “a slip may or may not occur.” Hence, participants were not aware of the sliders’ condition (e.g., unlocked, or locked) in all the trials. The starting position was randomly adjusted to allow 2–4 steps before landing on the right slider, so the participants were not aware when (at which step) the slip would occur. The instruction and the design of experimental setup could minimize participants’ anticipation of the upcoming trial (slip occurrence and timing).

It is widely known that a single slip trial exposure is sufficient to induce changes in reactive balance, which is known as the first-trial effect^23,33^. After a single slip exposure, individuals show significant improvements in stability and a reduction in the slip distance on the second slip, even though a majority (> 80%) still lose their balance^11^. Thus, only the first trial was used to represent the true novel slip response (no-adaptation). Participants rapidly adapted within 3 to 5 slip perturbations, represented by the great improvement in slip outcomes (from balance loss to no balance loss)^34^. Therefore, S6–S8 were selected to represent the early-adaptation stage. The last three trials (S22–S24) following 3 blocks of slip training were used for the late-adaptation stage in this study.

### Data collection and processing

Subjects were protected by a full-body harness connected to a loadcell (Transcell Technology Inc., Buffalo Grove, IL) by shock-absorbing ropes. Four force plates (AMTI, Newton, MA) were installed beneath the walkway, including two force plates on each side of the walkway. There was no contact between the right and left side of the walkway and participants were instructed to walk without crossing the midline between left and right sides. The force plates recorded the vertical ground reaction force from leading foot touchdown to up to 4 steps after slip onset. Once a participant’s right (slipping) foot was detected by the force plates, a computer controlled triggering mechanism would release the right platform^9^. Kinematics from a modified Helen Hayes marker set (26 retro-reflective markers)^35^ were recorded by an 8-camera motion capture system (Motion Analysis Corporation, Santa Rosa, CA). The 26 markers were placed on vertex, ears, rear neck, shoulders, midpoint of the right scapula, elbows, wrists, sacrum, greater trochanters, mid-thighs, knees, mid-shanks, ankles, heels, and the fifth metatarsal heads. Another two markers were placed on the sliders^36^.

The wired sliver-sliver chloride surface electrodes (1 cm diameter and 2 cm inter-electrode distance) with inbuilt preamplifiers of gain × 35 (EQ Inc, Chalfont, PA) were used to record surface electromyography (EMG) activity from 4 muscles on each leg at 600 Hz. The ground electrode was placed on the anterior superior iliac spine. EMG signals from the preamplifiers were filtered (fourth-order low-pass Bessel filter with a 300 Hz cutoff frequency) and amplified (gain × 100) with a CyberAmp 380 amplifier (Axon Instruments, Union City, CA). The EMG data, loadcell data, and force plate data were collected by the same data acquisition system at 600 Hz, which was synchronized with the kinematic data collected at 120 Hz.

EMG was recorded from the tibialis anterior (TA), medial gastrocnemius (MGAS), vastus lateralis (VLAT), and biceps femoris long head (BFLH). Using custom MATLAB (MathWorks, Natick, MA) routines, raw, unrectified EMG signals were digital high-pass filtered at 35 Hz after data collection and then full-wave rectified. The rectified data was smoothed via a second-order, dual-pass (both forward and backward directions) Butterworth low-pass filter with a 40 Hz cutoff frequency^20^.

### Outcome variables

#### Gait-related variables

The crucial time events were left foot touchdown (LTD1) prior to slip-onset, slipping (always right) foot touchdown (RTD, around 30 ms before slip-onset)^37^, post-slip recovery (always left) foot lift-off (LLO), and recovery foot touchdown (LTD2) in slip trials. These events were detected from the vertical ground reaction force (GRF decent below 10 N). Post-slip aborted steps taken by two participants, characterized by an unloading of the trailing limb upon slip onset, then reloading before its complete unloading were noted. As the GRF in these trials never decent below 10 N during the unload/reload period, their time events (LLO and LTD2) were detected based on the instant of minimum GRF^37^. Slip onset was defined as the time when the velocity of the slider marker exceeded 0.05 m/s in anteroposterior (AP) direction^38^. The duration analyzed in this study was between LTD1 and LTD2, which is one gait cycle. This duration contains both proactive phase prior to slip-onset and reactive phase after slip-onset. The duration of recovery foot swing phase was estimated by subtracting LLO from LTD2. COM position was calculated as the distance between the projected COM location (estimated using a 13-segment model)^39^ and slipping heel at pre-LTD2 (~ 10 ms prior to LTD2) in AP direction. COM velocity was calculated by subtracting the velocity of slipping foot (heel) from the velocity of COM at pre-LTD2. Dynamic stability was then calculated was the shortest distance from the instantaneous COM state (normalized COM position and velocity) to thresholds against backward balance loss^39^. Positive stability indicate a stable COM state with a lower likelihood of backward balance loss, while negative value indicates an unstable state with a higher likelihood of balance loss. Here the pre-LTD2 was chosen as the stability at this instant has been proved to determine the slip outcome^40^. Hip height was calculated as the midpoint of hip markers in the vertical plane at LTD2, which could quantify the limb support. Slipping distance was calculated as the maximum displacement of the slider marker from the touchdown of slipping foot (RTD) to its lift-off, and slipping velocity was the maximum velocity of the slider in this duration. Step length was calculated as the heel distance between both limbs at LTD2, and then normalized by foot length. The trunk angle was calculated as the angle between the trunk segment and the horizontal plane, and the maximum trunk angle was calculated as the peak value between RTD and LTD2. All these variables were calculated in the sagittal plane and could be considered as good representatives of slip adaptation.

#### Slip outcomes

Slip outcomes were classified as a backward loss of balance (BLOB) or no backward loss of balance (NLOB). Slip outcome was classified as a BLOB when the recovery heel landed posterior to the sliding heel^11^, or NLOB when the recovery heel landed anterior to the sliding heel and recovery stepping was not needed.

#### Muscle synergy extraction

EMG data for 8 muscles from LTD1 to LTD2 was down-sampled by averaging the data in 30 ms bins, and then EMG data from each trial was concatenated end-to-end to create matrices that were 8 (number of muscles) × n (number of time bins) in size^21^. The small number of muscles (n = 8) recorded for this study might lead to an underestimation of the number of muscle synergies^41^. The 30 ms time bins were selected in order to have sufficient time resolution and to avoid dealing with point to point changes in the highly variable EMG signals^42^. In addition, our previous study has compared two different time bins and found that there was very limited effect of time bins on the structure of muscle synergies^43^.

Due to the fast adaptation in kinematic outcomes to slip perturbations, changes in neuromuscular control could also occur after very few slips^23^. Therefore, only the first slip trial was used for muscle synergy extraction for the novel condition. A prior study has reported increased intra-subject variability among the muscle synergies extracted from single trial compared to concatenated trials, suggesting multiple trials are more reliable to capture the inherent EMG variability representing motor pattern^44^. Therefore, three consecutive trials in each stage were used for muscle synergy extraction for early-adaptation and late-adaptation stages, and these trials were simply concatenated end-to-end in each stage. The single and concatenated EMG matrices were then normalized to the maximum activation in all non-slip trials prior to the novel slip trial^45^, and each row (muscle vector) was scaled to have unit variance to ensure that each muscle was equally weighted in the extraction. This unit variance was removed after muscle synergy extraction, changing the muscle synergies back to their original scaling.

Muscle synergies were extracted from EMG matrices with unit variance for each condition (novel, early-, and late-adaptation) by non-negative matrix factorization using customized Matlab routines^46^, which is a decomposition algorithm used extensively in muscle synergy analysis^21,45,47^. This algorithm assumes that a muscle activation pattern, M, which was evoked by a perturbation (slip or auditory cue) in a given time period, is comprised of a linear combination of a few muscle synergies, *w*_*i*_, which are each recruited by a synergy recruitment coefficient, *c*_*i*_. Therefore, a particular muscle activation pattern would be characterized by the following equation: M = *c*_*1*_* w*_*1*_ + *c*_*2*_* w*_*2*_ + ⋯ + *c*_*n*_* w*_*n*_ + ***ε***.

In this equation, ***ε*** is a scalar of EMG noise, *w*_*i*_ is a vector corresponding to the *i*th muscle synergy, and each *w*_*i*_ is multiplied by a scalar recruitment coefficient, *c*_*i*_. The spatial components were considered a fixed time-invariant pattern, while the temporal activation coefficients varied across time^48^; therefore, the activation represents how the group of muscles in *w* is activated over time. If the average contribution of one component in a muscle synergy was above the threshold of 0.4, it was considered as a major contributor to that synergy^49^. In order to compare the temporal activation coefficients among different stages, these coefficients in one gait cycle (LTD1 to LTD2) were scaled to same length for each subject with 100 points.

The number of muscle synergies required to explain any of these datasets was determined by selecting the smallest number of synergies that could adequately reconstruct the muscle responses, which was quantified by the variance accounted for (VAF). To ensure consistency in selecting the number of muscle synergies within each condition, the number of muscle synergies selected was the minimum number at which the muscle synergies accounted for greater than 75% of the VAF in each muscle and exceeded 90% of the overall VAF^20^. These VAF thresholds could guarantee that the recorded muscle curves could be well reconstructed.

To facilitate the comparison of muscle coordination patterns between stages, muscle synergies extracted from each condition were firstly pooled across subjects and grouped with a hierarchical cluster analysis^21^. Such method could also reduce the effect of the innate variability of the muscle activity on the extracted motor patterns. The number of clusters for each condition was determined by identifying the minimum number of clusters that partitioned the muscle synergies such that no cluster contained more than one muscle synergy from the same subject.

#### Modification of muscle synergies

**Figure 1:**
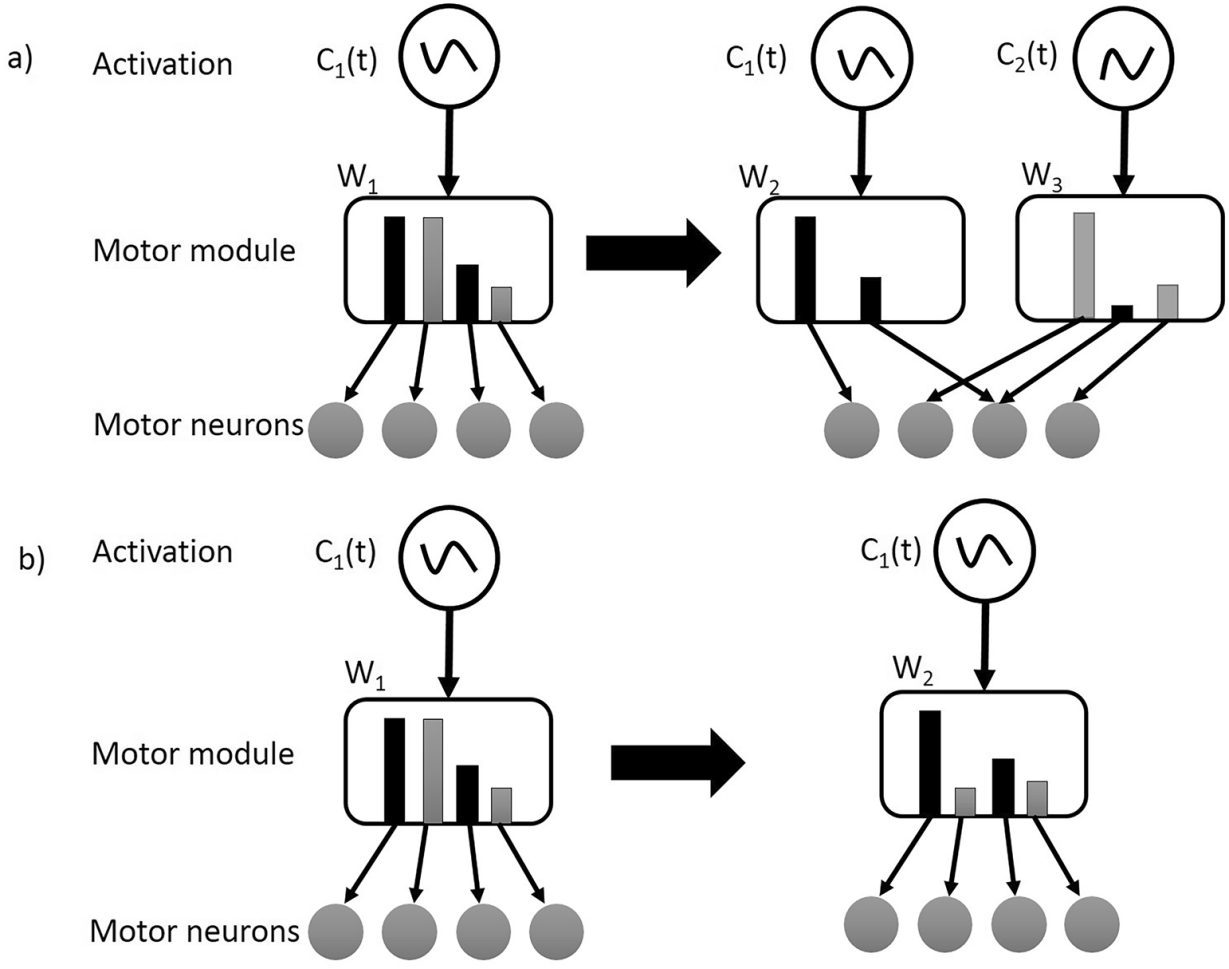
Schematic diagram of the muscle-synergy generation through a) fractionating the pre-existing synergies and b) modifying the weight(s) of pre-existing synergies. Fractionation of synergies could be attributed to the change of oscillator, which generates burst activities at different phases of the gait cycle. The modification of synergies is only related to the synergy-encoding interneurons, which determines the structure of synergies.

Previous studies have shown that the CNS could modify existing motor patterns to adapt to different perturbations, including merging, fractionation, and adjustments of weight(s) in the structure of muscle synergy^50–53^. The merging of muscle synergies could be achieved by reassigning multiple synergy-encoding interneurons to be driven by the same oscillator. Conversely, the fractionation could be achieved by reassigning the synergy-encoding interneurons to be driven by a different oscillator (Fig. 1a). The adjustments of muscle synergy only changed the weight(s) in the synergy-encoding interneurons but not the oscillator (Fig. 1b). To verify whether there was merging and fractionation of muscle synergies during slip training, each clustered synergy was modeled as a linear combination of the set of clustered synergies from the other stage^54^. The coefficients of this linear combination were identified through a nonnegative least-squares procedure. If a single synergy from one stage was similar to the combination of a set of synergies from another stage (based on their correlation coefficient), this single synergy would be considered as the merging of the set of synergies, while the set of synergies could be also considered as the fractionation of the single synergy. To analyze the adjustments of weight(s) in the structure of a muscle synergy, the weights were compared between different synergies with similar activation coefficients.

#### Cross validation of the clustered muscle synergies

To verify the robustness of the clustered muscle synergies (the consistency of cluster numbers across different samples), we used a cross-validation procedure^55,56^. Five random subjects were removed from the whole dataset, and then the muscle synergies were extracted from this sub-maximum (25/30) dataset for no-adaptation stage, early-adaptation stage, and late-adaptation stage separately. This process was repeatedly conducted 300 times, and the averaged cluster numbers for each stage were calculated. In addition, we also compared the similarity (r value) between the clustered muscle synergies from a random selected sub-dataset and the one from the whole dataset for each stage.

### Statistical analysis

To investigate the kinematic differences in different adaptation stages (novel slip, early-, and late-adaptation), normal distribution was checked in advance using Kolmogorov–Smirnov test. Kruskal–Wallis test was conducted for the variables with non-normal distribution (rate of BLOB), and repeated ANOVA was conducted to compare adaptation effects for the normally distributed variables (COM position, COM velocity, stability, step length, swing phase, hip height, maximum slipping distance, maximum slipping velocity, and maximum trunk angle). Mauchly’s sphericity test was used to validate the repeated ANOVA, and Huynh–Feldt correction was conducted when sphericity was violated. Lastly, significant main effects were resolved using paired t-tests between groups. Benjamini and Yekutieli (B-Y) corrections were applied to adjust the false discovery rate for these Post hoc tests (corrected α = 0.024)^57^.

To test the differences in muscle synergies among different adaptation stages, the number of muscle synergies were first compared using a paired Wilcoxon signed rank test. Cohen’s d mean difference effect sizes and power sizes were also calculated to verify the validity of these results. Next, the correlation coefficient (r) was calculated between the average muscle synergy vectors and between the average recruitment coefficients from the clusters to compare the coordination pattern of muscle synergies. A pair of muscle synergy vectors (degree of freedom is 6) were considered similar if they had r > 0.834, which represents statistically significant similarity (p < 0.01)^18^. The muscle synergies from different stages with significant similarity were classified into the same mode. For the recruitment coefficients (degrees of freedom = 98), the similarity was checked for those muscle synergies from the same mode, and any pair with r > 0.3 was considered to be significantly similar (p < 0.01).

## Results

### Kinematic changes during repeated slip training

During the slip perturbation training, all 30 participants lost their balance on novel slip trials (S1), but successfully recovered in the subsequent early-adaptation trials (S6–S8) and late-adaptation trials (S22–S24) with a BLOB rate of 0% for both phases. Only two participants demonstrated an aborted stepping response while the rest all took a backward recovery step. The repeated ANOVA results indicated that there was a significant training effect on BLOB rate (p < 0.001, Table 1). Consistently, a significant training effect was found on all the kinematic variables (p < 0.001 for all, Table 1). Post hoc tests indicated that these kinematic variables were significantly better (greater COM position and velocity, larger stability and step length, longer swing phase, higher hip height, and lower slip intensity) in the early- and late-adaptation trials than the no-adaptation trials (p ≤ 0.01 for all).

**Table 1:** Repeated ANOVA results of all the gait variables during slipping (*F* value) and Kruskal–Wallis test for BLOB rate (*χ*^2^).

Variable	Mean ± SD			Mauchly	repeated ANOVA	
	NS	EA	LA	*P*	*P*	*F/χ*^*2*^
BLOB	100%*	0%	0%	Nan	< 0.001	> 1000
COMx (m)	− 0.22 ± 0.08*	0.36 ± 0.12	0.34 ± 0.1	> 0.05	< 0.001	358.8
COMv (m/s)	− 0.66 ± 0.62*	1.23 ± 0.49	1.16 ± 0.38	> 0.05	< 0.001	144.6
Stability	− 0.51 ± 0.2*	0.81 ± 0.21	0.79 ± 0.19	> 0.05	< 0.001	632.8
SL (m)	− 0.52 ± 0.17*	0.53 ± 0.21	0.5 ± 0.18	> 0.05	< 0.001	337.6
Slide_x (m)	0.67 ± 0.23*	0.08 ± 0.08	0.11 ± 0.12	< 0.001	< 0.001	172.9
Slide_v (m/s)	2.11 ± 0.40*	0.38 ± 0.21	0.43 ± 0.31	0.03	< 0.001	312.6
Swing time(s)	0.13 ± 0.06*	0.37 ± 0.06	0.38 ± 0.07	< 0.001	< 0.001	180.2
Recovery time(s)	0.33 ± 0.06*	0.6 ± 0.10	0.59 ± 0.08	< 0.001	< 0.001	204.6
Hip height (m)	0.77 ± 0.07^#^	0.78 ± 0.07	0.78 ± 0.07	< 0.001	0.003	7
Trunk (deg)	93.9 ± 5.0*	89.8 ± 3.8	90.3 ± 4.0	> 0.05	< 0.001	24.3

Positive stability indicate stable COM state, and negative value indicate unstable COM state. Trunk angle > 90 indicates trunk extension, and the angle < 90 indicates trunk flexion. Recovery time is the duration between RTD and LTD2, COMx, COMv, and stability was calculated at pre-LTD2. Slide_x denotes slip distance, slide_v denotes slip velocity, NS denotes novel slip, EA denotes early-adaptation, and LA denotes late-adaptation.

*Significant difference between NS and EA, and between NS and LA (p < 0.001 for all).

^#^Significant difference between NS and EA, and between NS and LA (p ≤ 0.01).

### Change in muscle synergies during slip adaptation

**Figure 2:**
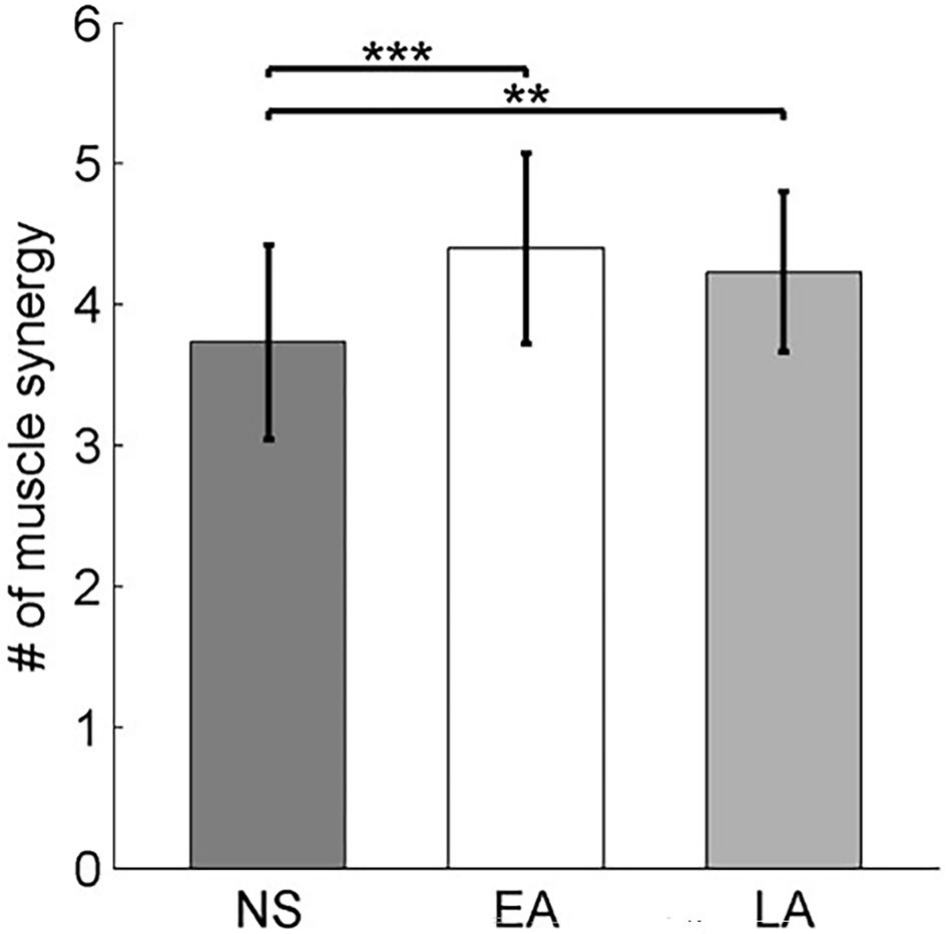
Number of muscle synergies (mean and SD) recruited for the novel-slip (dark gray bar), early-adaptation (white bar), and late-adaptation stages (light gray bar). ** denotes p < 0.01, *** denotes p < 0.001.

After receiving the slip training, participants tended to use more muscle synergies in the adapted slips compared to their novel slips, represented by a significant increment in the muscle synergy number from no-adaptation slips (n = 3.7 ± 0.7) to the early-adaptation (n = 4.4 ± 0.7) and late-adaptation (n = 4.2 ± 0.6) slips (p < 0.01 for both, Fig. 2). However, no difference in the muscle synergy numbers was found between the two adaptation stages.

**Figure 3:**
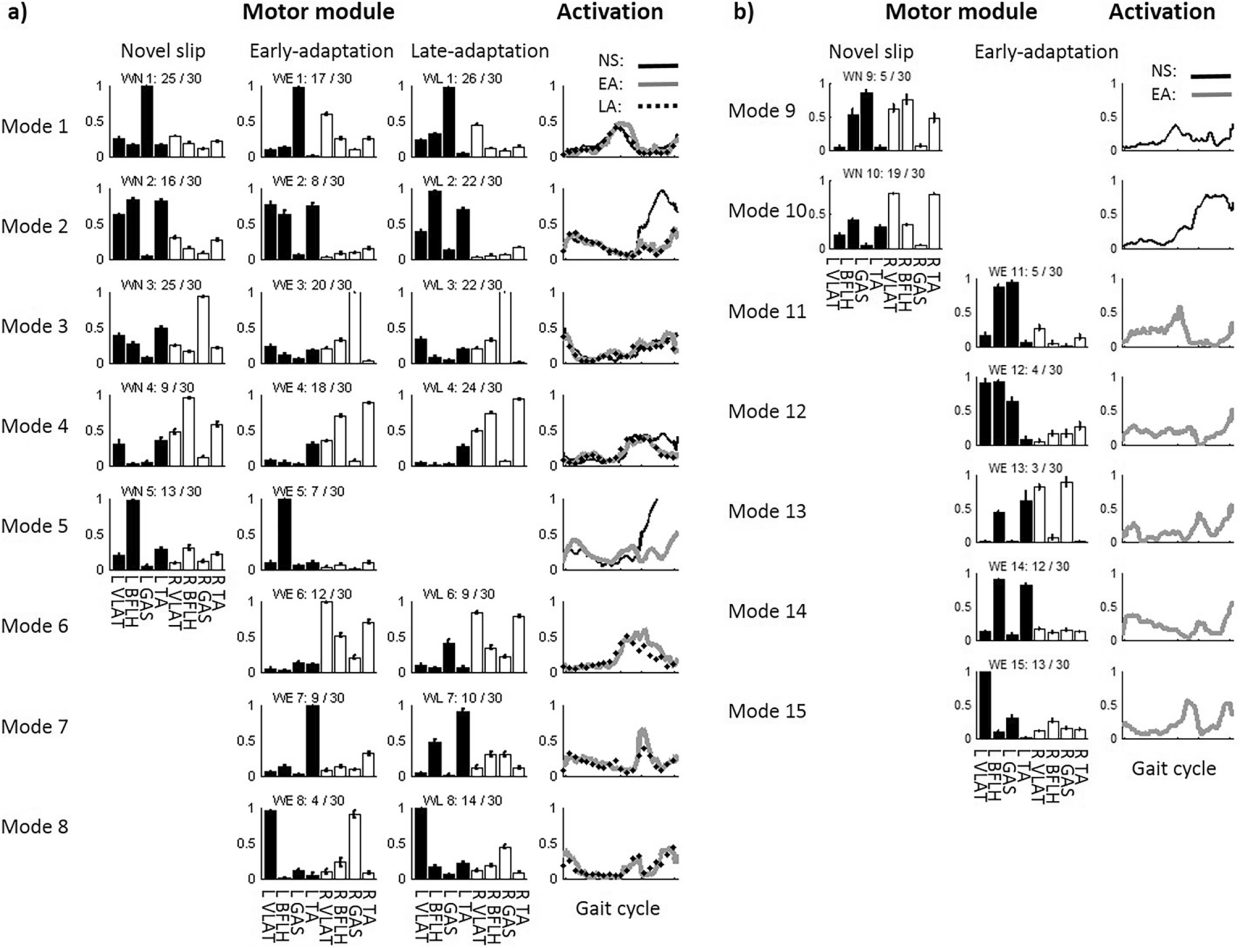
(**a**) The common muscle synergy clusters (Modes 1–8) during repeated slip training. (**b**) The unique muscle synergy clusters (Modes 9–15) during repeated slip training. It should be noted that no unique muscle synergy was detected in the late-adaptation stage. Mean values are represented by the bars in the motor module (matrix *w* in the synergy equation) figure and by the black lines in the temporal activation (matrix *c* in the synergy equation) figure. Standard error (SE) is represented by the error bars in the motor module. The first 4 muscles in each muscle synergy are for the recovery limb (L) and the other 4 are for the stance limb (R). x/30 adjacent to each muscle synergy indicates x out of 30 participants recruited that muscle synergy. WNi denotes muscle synergy during novel slips, WEi denotes muscle synergy during early-adaptation stage, WLi denotes muscle synergy during late-adaptation stage, and i denotes which mode they belong to.

The structure of the muscle synergies also changed along with the slip perturbation training. The muscle synergies for all three stages could be classified into 15 different modes based on the synergy structure. Specifically, 7 modes were recruited in novel slips (WN*i*), 13 were recruited in early-adaptation (WE*i*), and 7 were recruited in late-adaptation (WL*i*). Here, *i* indicates which mode the synergy belongs to (e.g., WN2 represents a synergy belonging to Mode 2 used in novel slips). Among these modes, Modes 1–8 were the common modes which were recruited by at least two different stages (Fig. 3a), while Modes 9–15 were the unique modes which only appeared in a single stage (Fig. 3b). Specifically, Modes 9–10 were only recruited in novel slip trials, Modes 11–15 were only recruited in early-adaptation trials, and none of these unique modes were recruited in the late-adaptation stage.

For the 8 common modes of muscle synergies, 4 of them were recruited throughout all three stages (Modes 1–4 in Tables 2 and 3). Mode 5 was recruited only in novel and early-adaptation slips, and Modes 6–8 were recruited in both adaptation stages. For their temporal activations, most of them also showed consistency in the activation curves (r > 0.67, Table 3). Only Modes 2 and 5 showed low similarity between novel slips and adapted slips (r < 0.36). The peak magnitude of the averaged activation curve for these two modes were much higher in the novel slips than the adapted slips, especially in the step execution period (Fig. 3).

**Table 2:** Similarity between the structure of muscle synergies across the novel slips (NS), early-adapted slips (EA), and late-adapted slips (LA).

Mode	NS	EA	r value	NS	LA	r value	EA	LA	r value
1	WN1	WE1	0.887	WN1	WL1	0.938	WE1	WL1	0.922
2	WN2	WE2	0.918	WN2	WL2	0.840	WE2	WL2	0.918
3	WN3	WE3	0.871	WN3	WL3	0.878	WE3	WL3	0.992
4	WN4	WE4	0.847	WN4	WL4	0.851	WE4	WL4	0.989
5	WN5	WE5	0.968						
6							WE6	WL6	0.916
7							WE7	WL7	0.847
8							WE8	WL8	0.870

The pairs with similarity > 0.834 (corresponding to p < 0.01) were classified into same mode, and total 8 modes were detected in this study.

**Table 3:** Similarity between the activation of across the novel slips (NS), early-adapted slips (EA), and late-adapted slips (LA) for each mode in common (Mode 1–8).

Mode	NS	EA	r value	NS	LA	r value	EA	LA	r value
1	CN1	CE1	0.824	CN1	CL1	0.945	CE1	CL1	0.926
2	CN2	CE2	0.258	CN2	CL2	0.190	CE2	CL2	0.918
3	CN3	CE3	0.812	CN3	CL3	0.793	CL3	CL3	0.894
4	CN4	CE4	0.777	CN4	CL4	0.676	CE4	CL4	0.950
5	CN5	CE5	0.352						
6							CE6	CL6	0.825
7							CE7	CL7	0.748
8							CE8	CL8	0.853

### Synergy changes from no-adaptation stage to early-adaptation stage

**Figure 4:**
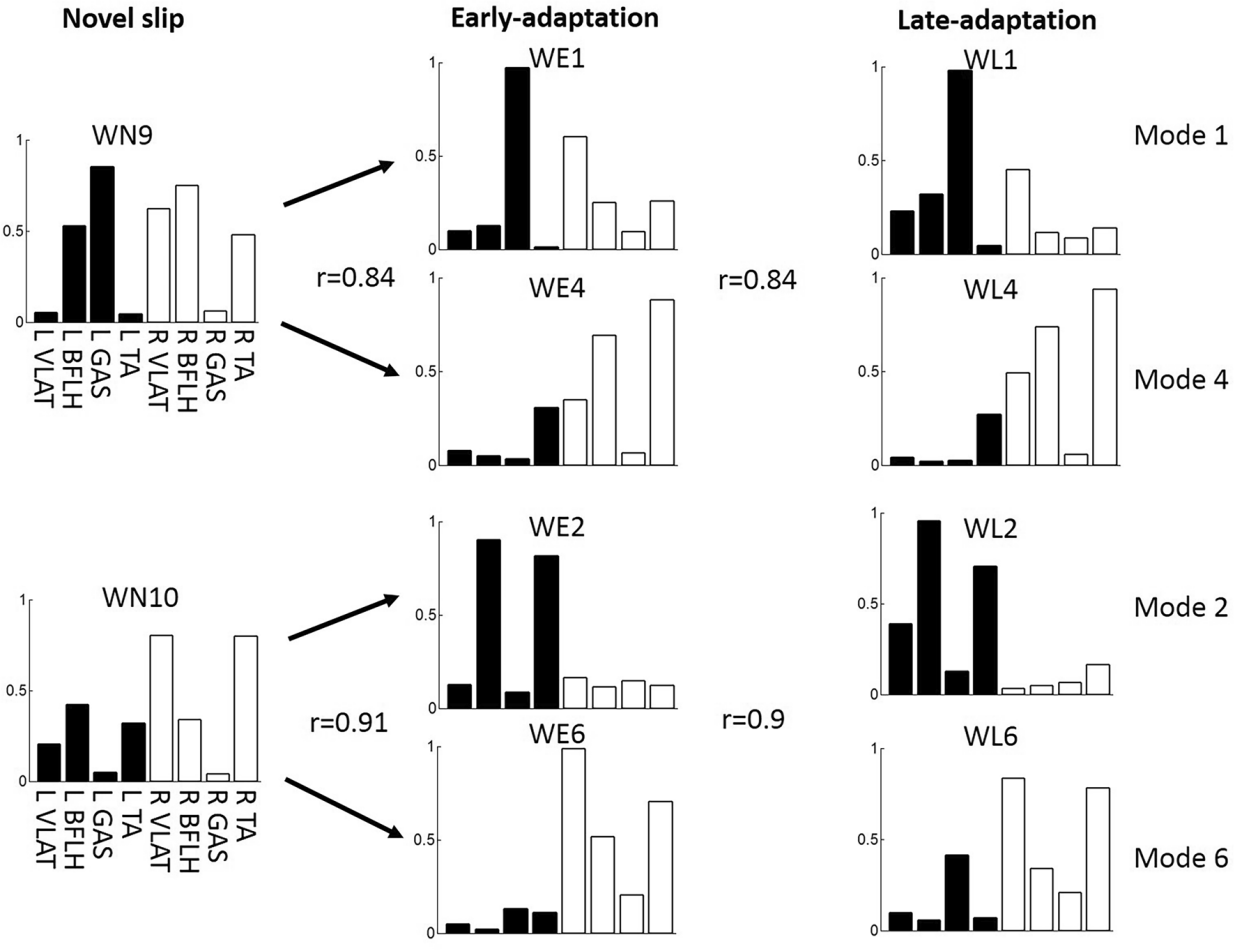
The fractionation (or merging) of muscle synergies. WN9 could be considered as the linear combination of WE1 and WE4 in the early-adaptation stage, or the combination of WL1 and WL4 in the late-adaptation stage. WN10 could be considered as the linear combination of WE2 and WE6 in the early-adaptation stage, or the combination of WL2 and WfL6 in the late-adaptation stage.

**Figure 5:**
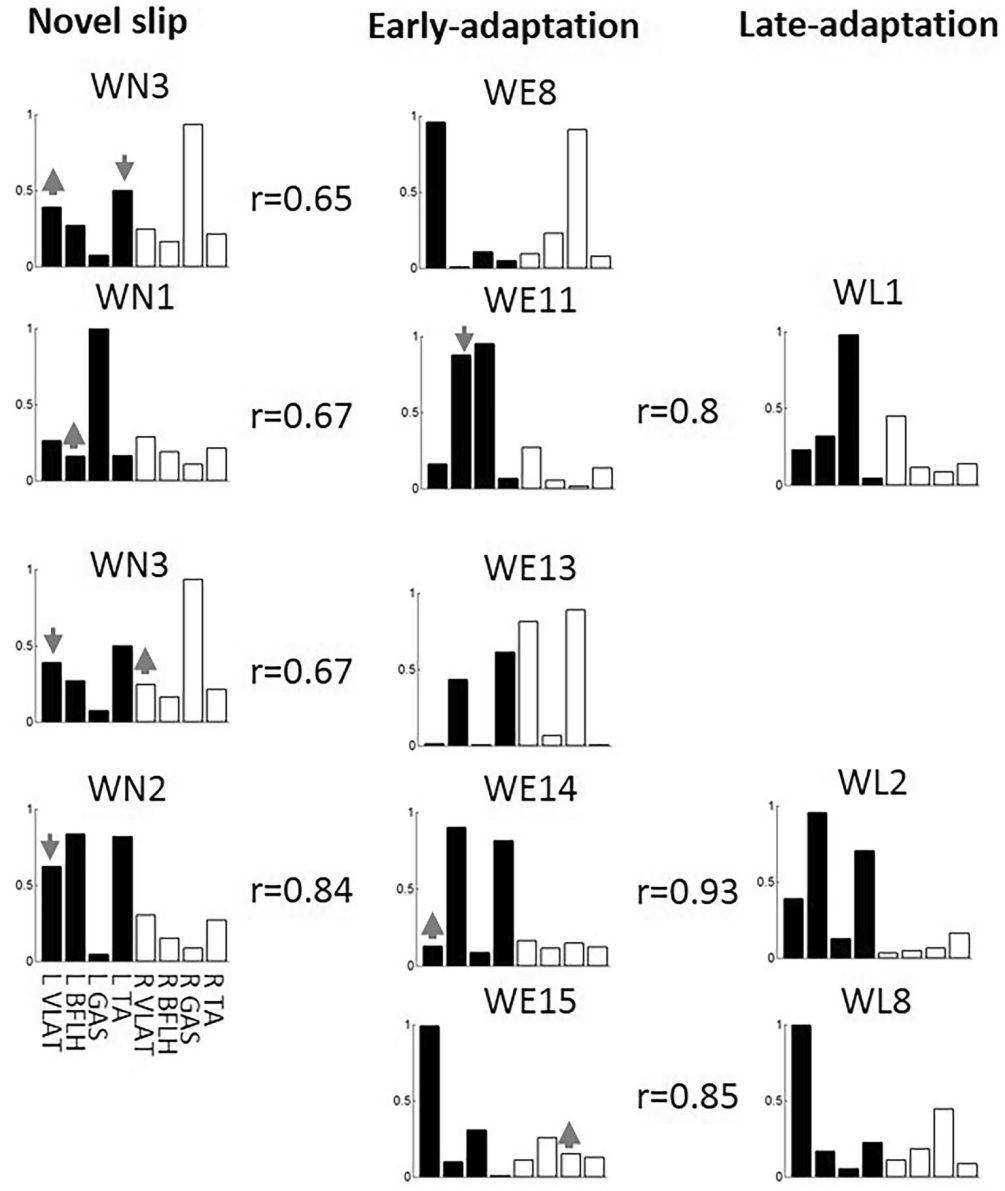
The generation of new muscle synergies in early-adaptation stage after receiving a block of slip training, and their changes in the late-adaptation stage after receiving three blocks of slip training.

Mode 9 and Mode 10 were only recruited in the novel slips but not in the adapted slips. As they showed similarity to the combination of the common muscle synergies (Modes 1–8), they were considered to be fractionated into units with fewer muscles in the early-adaptation stage. Specifically, Mode 9 was fractionated into Mode 1 and Mode 4, and Mode 10 was fractionated into Mode 2 and Mode 6 (Fig. 4). Besides the fractionated one (Mode 6), there were another seven new synergy modes (WE7, WE8, and WE11–15) not used in the novel slips. We note that the structure of WE14 was similar to WN2 in novel slips but not WE2 in early-adaptation stage, while the temporal activations for these three synergies are highly consistent; hence, WE14 could be considered as a modified version of WN2 (or Mode 2) by lowering the weight of muscle VLAT in recovery limb (Fig. 5). Similarly, WE11 showed a moderate similarity in structure and high similarity in activation to WN1; therefore, it could also be considered as a modified version of WN1 by increasing the weight of BFLH in recovery limb, and WE13 could be considered a modified of WN3 by adjusting the weight of both VLAT muscles (Fig. 5).

### Synergy changes from early-adaptation stage to late-adaptation stage

The mode of muscle synergies decreased from 13 to 7 in this stage, and all the 7 muscle synergies recruited in the late-adaptation stage had appeared in the early-adaptation stage (Fig. 3). Among the 8 new synergies generated in the early-adaptation stage, only Modes 6, 7, and 8 were retained, and the discarded synergies (Modes 9–15) seemed to be redundant. For example, only slight differences in the structure were found between WE8 and WE15, and both were similar to WL8 in structure and activation.

### Cross-validation

**Figure 6:**
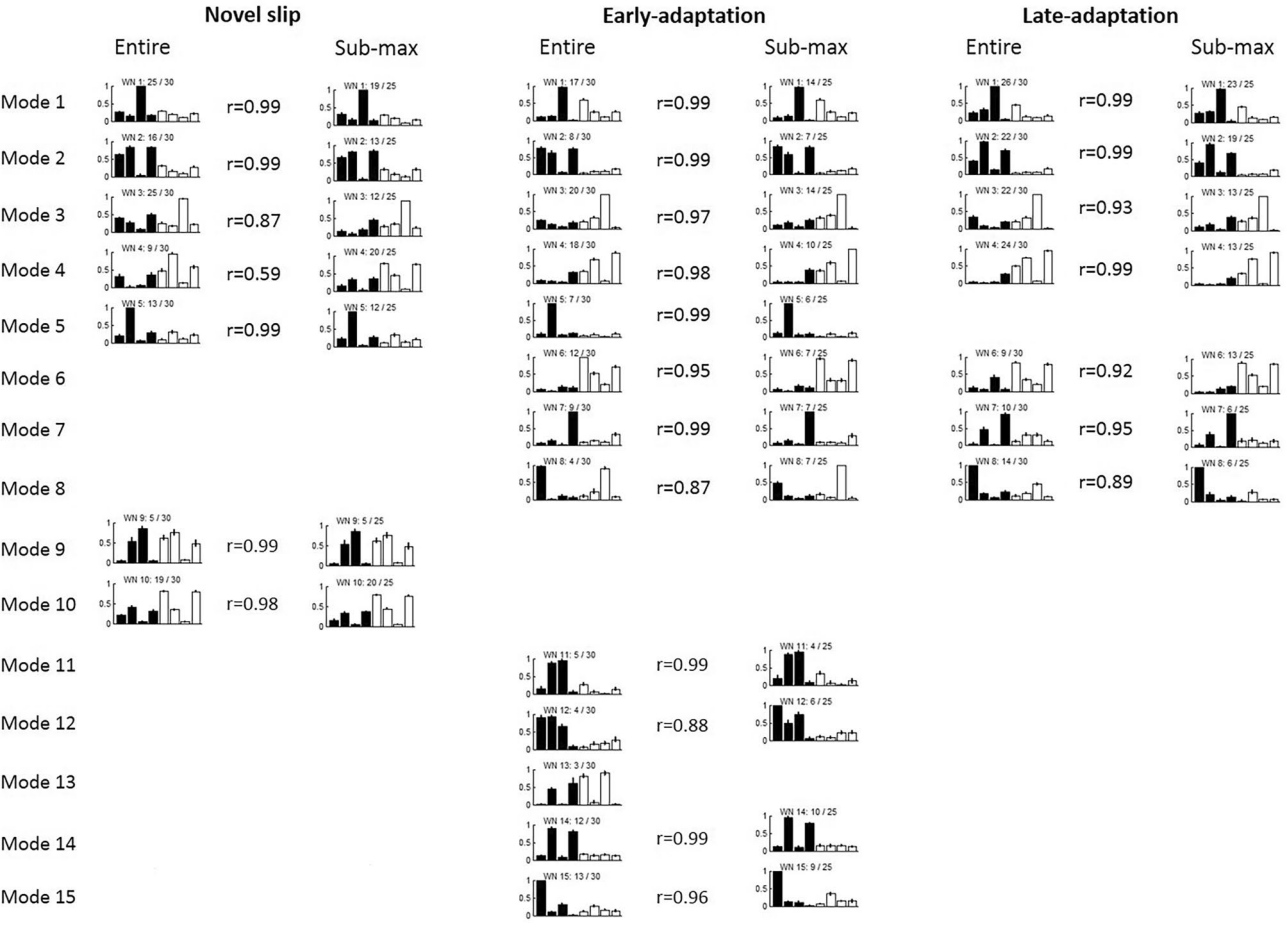
The comparison of clustered muscle synergies between entire dataset and sub-maximum (25 out of 30) dataset.

The cross-validation of clustered results indicated that the number of clustered synergies (n = 9.4 ± 3 for no-adaptation, n = 12.8 ± 2.4 for early-adaptation, and n = 8.4 ± 1.4 for late-adaptation) from the sub-maximum dataset were similar with those extracted from the entire dataset (n = 7, 13, and 7 for these three stages). More importantly, even though there was a slight change in the number of clusters after reducing the sample size, the synergy modes were highly consistent with those extracted from the entire dataset (r ≥ 0.87 for 25 out of 27 clustered muscle synergies, Fig. 6).

## Discussion

This study firstly revealed that although majority of the muscle synergies (5 out of 7) used in novel slips are similar to those used in the adapted trials, there are significant changes in neuromuscular activity during repeated slip training in healthy older adults. Following repeated training, specific patterns of muscle synergies emerged to possibly compensate for the sensorimotor errors due to external perturbation. Around half of these new muscle synergy modes in the early-adaptation stage did not appear in the late-adaptation stage, indicating patterns of muscle synergies also significantly changed in the late-adaptation stage.

Kinematic results indicated that all the participants rapidly adapted to the slip perturbation within 8 trials (early-adaptation stage), which was consistent with the previous findings^4,58^. Significant changes were detected from the novel slip to the adapted slips (S6–S8) in the step length, gait phase, stability, limb support (hip height), and the control of slip intensity. As a part of adaptation, participants lowered the slip perturbation intensity (displacement and velocity) and increased their swing phase which could have allowed them to take a longer recovery step. Such kinematic changes resulted in the BLOB rate dropping from 100% on S1 to 0% on S6–S8. Even if larger stability could increase the risk of forward balance loss, a previous study reported that prior adaptation to slips had only limited interference during the initial phase of trip recovery^59^, suggesting that the slip adaptation probably would not lead to forward balance loss. There was no change in kinematic variables between early-and late-adaptation phase. This suggests that larger training dosage may not serve to further improve gait characteristics and COM stability, while it is still possible that a larger training dosage might keep fine-tuning or optimizing the individual joints and their coordination, which could not be represented by gait kinematics and stability.

The majority of muscle synergies (5 out of 7) remained the same across the novel slip as well as early- and late-adaptation stages. Even though the other two muscle synergies (Modes 9 and 10, Fig. 3b) only appeared in the novel slips, they could be considered as combined synergies from the common modes. Specifically, Mode 9 was the linear combination of Mode 1 and Mode 4, and Mode 10 was the combination of Mode 2 and Mode 6 (Fig. 4). Such combination resulted in a fewer number of muscle synergies recruited by each participant in their novel slips compared to the adapted ones (Fig. 2). It is postulated that the number of muscle synergies used by the CNS are related to complexity in movement control^60^. As the novel slips always had a higher slip perturbation intensity (longer slip distance and faster slip velocity in Table 1), the speed of the reactive response is critical. To compensate for the time constraints during a novel slip (shorter duration between slipping TD and recovery TD), participants might reduce the complexity of motor control, resulting in the combination (merging) of muscle synergies and reduction of muscle synergy number. The combination of muscle synergies is proposed to be accomplished through assigning the synergy-encoding interneurons to be driven by the same oscillator, which generates burst activities at different phases of the gait cycle^61,62^. In other words, the structure of synergies did not change, only their temporal activity changed during adaptation, as shown in Fig. 1a. Therefore, our results were consistent with the previous findings demonstrating that majority of muscle synergies were common between different postural tasks^18,20^, indicating that a generalized motor program might be used for balance control across different contexts.

Our results indicated that the combined (merged) synergies were only recruited in the novel slips, as such strategy could not lower the slip intensity and prevent backward balance loss^11^. Hence, after receiving training, none of these combined synergies appeared again. The combined synergies were fractionated into units with fewer muscles (Modes 1, 2, 4, and 6) within only 6 repeated trials, suggesting that repeated perturbation training is an effective method for the fractionation of muscle synergies. This finding was consistent with previous arguments that the fractionation of muscle synergies is a process primarily driven by development and training^62^. The fractionation of muscle synergies in the adapted trials indicated that participants changed their recovery strategies for balance control, at least by stopping reliance on quick recovery stepping for fall prevention in these repeated slips (time of recovery stepping increased by ~ 0.3 s, Table 1).

Our results also reflected that the CNS tends to modify existing motor patterns rather than assembling new patterns^62^, including fractionation, and adjustments of the weight for specific muscle(s). For the 4 new synergies (WE8, WE11, WE13, and WE14) generated from weight adjustments, most of the adjustments occurred on the recovery side, and 3 out of 4 synergies modified the weight of VLAT of the recovery limb. Specifically, the weight of VLAT in WE8 increased to the maximum level, which could improve the knee extensor moment after recovery touchdown and enhance the propulsive force required to improve COM stability and limb support^63^. While the weight of VLAT decreased to ~ 0 in WE13 and WE14, the temporal activation of these two synergies indicated that their peak occurred in the swing phase, suggesting that both synergies might help moving the swing leg anterirorly. Both synergies (WE13 and WE14) showed a co-activation of BFLH and TA, which could increase the knee flexion and dorsi-flexion of the swing leg, resulting in a longer recovery step and larger toe clearance. Similarly, the co-activation of BFLH and TA could also be seen in WL7, indicating that such strategy was retained after repeated slip training for taking a post-slip step. The adjustments of the motor pattern indicated that the CNS might adopt the optimization algorithm to search for the optimal synergies during slip adaptation in gait, and the weight of motor patterns might be adjusted heuristically based on previous experience^64^. However, there were 2 muscle synergies (WE12 and WE15) that showed low similarity to all the synergies used in novel slips. Both synergies showed a co-activation of VLAT and GAS in the recovery limb, which could lead to knee extensor and plantar flexion to initiate a foot lift-off. These synergies might be due to the inter-individual variability in slip adaptation, but it is also possible that these synergies might be generated from other pre-existing synergies which were either inborn or acquired during other trainings^65^. In general, a majority of the new synergies could be considered as altered motor programs (common synergies) for balance control, and this fine-tuning of neuromuscular control would greatly affect the slip outcomes.

The temporal activation of these generated synergies showed that some of them (e.g., Mode 6 and 8) were activated before slip-onset. These modes might be related to proactive adjustments, which can greatly lower slip intensity. Previous evidence has shown that slip intensity is highly related to changes in kinematics of the slipping limb ankle and knee joints^11,66^. The activation of the MGAS muscle in Mode 8 could lead to a flat foot landing, and the co-activation of the BFLH and VLAT in Mode 6 could increase the stiffness of the slipping limb knee joint. The reduction of the slip intensity could further improve the COM stability, and lower the likelihood of fall or backward balance loss. In addition, there are no muscle synergies in the novel slips that demonstrated activation of the slipping limb MGAS around slip onset, which might be one of the key factors leading to higher slip intensity in novel slips. All these findings suggested that there is a shift from a reliance on feedback control to feedforward control after repeated slip training, which is consistent with previous postulations^4,67,68^.

The CNS may not generate any new synergies in the late-adaptation stage after receiving a block of mixed-training trials, but only select the optimal ones or discard the redundant ones generated in the earlier stage. The principal reason to discard these redundant muscle synergies might be that they could affect efficiency of the gait pattern, if retained. For example, WE8 and WE15 were similar in both structure and activation. It can be postulated that such small differences might have had very limited effects on balance control and slip recovery outcomes. Hence, the discard of WE15 in the late-adaptation phase had very limited effects on the motor performance, represented by similar kinematic measures between early- and late-adaptation. Hence, it is possible that the redundant muscle synergies were abandoned in the late-adaptation phase. For WE12, although the coactivation of BFLH and VLAT in the recovery limb could enhance joint stiffness^69^ and result in higher stability and lower fall risk^70^, it could also reduce knee flexion in swing-phase^71^, leading to an abnormal gait pattern and higher energy cost. After experiencing the mixed-training block, participants may abandon this type of synergy, as the increment of stiffness (e.g., WE6) in the slipping limb alone might be sufficient for balance control. Such findings further support our postulation that an optimization algorithm might be used to change muscle synergies during adaptation. Our findings indicated that no new synergies were generated from early-adaptation to late-adaptation after the plateau in performance was reached (around S8). The only change between these two stages was the optimization of muscle synergies by discarding the redundant synergies. These findings were consistent with the previous postulation that providing more repetitions once the plateau in performance is reached could reduce redundancies and consolidate the motor response^72^. Our findings indicated that a single training session was sufficient to induce changes in muscle synergies; however, it is still unclear whether the generated synergies or optimized synergies could be retained in a long period. Until now, only short-term retention of the acquired muscle coordination patterns has been investigated and proved^73,74^, while previous studies have reported that the acquired motor patterns could be retained for over three months^31,75^. It is possible that the retained motor patterns were due to the long-term retention of altered muscle synergies, and such possibility deserves further analysis.

There are other factors which might affect the extraction of muscle synergies, such as the dominant side and muscle strength. As the perturbation was only triggered on the dominant side and the dominant side was found to have a higher number of extracted muscle synergies compared to the non-dominant side, it is possible that different synergy modes might be recruited on the non-dominant side compared to the dominant one^32^. However, the inter-limb difference in synergy number was only found in the upper limb movements, and there is no evidence indicating that lower limb movements also have the same inter-limb difference. Furthermore, a previous study found that the acquired motor strategies for fall prevention could be transferred from the training limb to the contralateral one^76^, suggesting that similar muscle synergies might be recruited between the dominant and non-dominant limbs for fall prevention. Therefore, we expect similar stage-to-stage changes should also be observed on the non-dominant side with just slight differences in the synergy modes. However, such expectation needs to be further verified. For the muscle strength of lower limbs, it has been reported that muscle weakness has limited influence on the extracted muscle synergies during gait^77,78^; hence, the lower limb strength would not affect our conclusion.

This study has certain limitations which have been considered while interpreting the results. This study only used one trial for analyzing the novel slip response compared to three trials each for the early and late phase. This was done as it is widely known that a single slip trial exposure is sufficient to induce adaptive changes – known as the first-trial effect^23,33^. Thus, using an average of the first three trials would contaminate the true novel slip response. Second, the number of muscles (n = 8) recorded for this study could be seen as another limitation of the study, and could lead to an underestimation of the number of muscle synergies recruited during walking and slip responses^41^. Although both the number of muscles and the choice of the muscles could impact the results of muscle synergies, the muscles included in the present analysis can be considered to be among the dominant muscles involved in the slipping response^21^. This may help offset the effect of a limited number of muscles being used in the muscle synergy analysis. For future studies, it is recommended to include data from more proximal muscles (hip and trunk) contributing to posture and balance control. Third, although our study tried to lower the anticipation of the upcoming slip, it is impossible to eliminate all the anticipation after the exposure of the novel slip. Thus, awareness of upcoming slip conditions might contribute to changes in muscle synergies, and the optimized synergies exhibited post-training might not be generalized to unexpected slips in real-life. However, previous reports have revealed that around 50% of real-life falls are induced by snowy or icy surfaces during winter^79,80^. Therefore, most slips experienced during daily living might not be totally unexpected when individuals expect they might experience a slip (e.g., when walking outside on a snowy or icy surface). Thus, our findings could at least generalize to parts of real-life situations. Lastly, a constant time bin of 30 ms was used in this study, as the duration of reactive stepping changed during the training session. Thus, the total number of time bins for each stage were different, which might affect the extracted muscle synergies. However, our previous study compared two different time bins and found that there was very limited effect of time bins on the structure of muscle synergies^43^. Further, no difference in the duration of reactive stepping was found between the early- and late- adaptation stage, and the selection of time bin would not affect our conclusion.

## Conclusion

In conclusion, our findings indicate that the CNS could generate effective muscle synergies through fractionating or modifying the pre-existing synergies recruited in the novel slips within 8 training trials. These new synergies could effectively compensate for the instability induced by the slip perturbation, but there were also redundant synergies generated in the early-adaptation stage. Because continued block and mixed training provides increased dosage, the CNS possibly chooses to retain optimal muscle synergies while the redundant ones are abandoned. Thus, our study provides evidence that the kinematic adaptation to overground slip-perturbations previously observed could be attributed to the recruitment of effective muscle synergies generated during repeated slip training. Our results suggest that the generation and elimination of muscle synergies is an important marker of neural change to adapt to external perturbations. The findings from our study could inform clinicians of basic mechanistic changes underlying slip outcome improvements and such knowledge could assist with designing perturbation-based slip training protocols for fall prevention.

## Abbreviations

CNS: Central nervous system
COM: Center of mass
EMG: Electromyography
VAF: Variance accounted for
TA: Tibialis anterior
MGAS: Medial gastrocnemius
VLAT: Vastus lateralis
BFLH: Biceps femoris long head
LTD1: Left foot touchdown prior to slip-onset
RTD: Slipping (always right) foot touchdown, immediately before slip-onset
LLO: Recovery (always left) foot lift-off
LTD2: Recovery foot touchdown
BLOB: Backward loss of balance
NLOB: No backward loss of balance
WNi: A synergy belongs to Mode *i* recruited in no-adaptation stage (novel slips)
WEi: A synergy belongs to Mode *i* recruited in early-adaptation stage
WLi: A synergy belongs to Mode *i* recruited in late-adaptation stage
